# Dynamic Analysis of Chlorophyll *a* Fluorescence in Response to Time-Variant Excitations during Strong Actinic Illumination and Application in Probing Plant Water Loss

**DOI:** 10.34133/plantphenomics.0151

**Published:** 2024-02-16

**Authors:** Junqing Chen, Ya Guo, Jinglu Tan

**Affiliations:** ^1^ Department of Chemical and Biomedical Engineering, University of Missouri, Columbia, MO, USA.; ^2^ School of IOT, Jiangnan University, Wuxi, Jiangsu, China.

## Abstract

Magnitude measurement of chlorophyll *a* fluorescence (ChlF) involves challenges, and dynamic responses to variable excitations may offer an alternative. In this research, ChlF was measured during strong actinic light by using a pseudo-random binary sequence as a time-variant multiple-frequency illumination excitation. The responses were observed in the time domain but were primarily analyzed in the frequency domain in terms of amplitude gain variations. The excitation amplitude was varied, and moisture loss was used to induce changes in the plant samples for further analysis. The results show that when nonphotochemical quenching (NPQ) activities start, the amplitude of ChlF responses vary, making the ChlF responses to illumination excitations nonlinear and nonstationary. NPQ influences the ChlF responses in low frequencies, most notably below 0.03 rad/s. The low-frequency gain is linearly correlated with NPQ and can thus be used as a reference to compensate for the variations in ChlF measurements. The high-frequency amplitude gain showed a stronger correlation with moisture loss after correction with the low-frequency gain. This work demonstrates the usefulness of dynamic characteristics in broadening the applications of ChlF measurements in plant analysis and offers a way to mitigate variabilities in ChlF measurements during strong actinic illumination.

## Introduction

Chlorophyll *a* fluorescence (ChlF) has been extensively used as a measurable signal to probe the photochemical reactions in plants [1]. The commonly used ChlF-based measures are computed from measured ChlF magnitudes (absolute levels) in response to an actinic light or saturating pulse. There are, however, inherent uncertainties in scaling and measuring the absolute magnitude of a quantity such as ChlF, which impact the usefulness of the existing method.

When a leaf receives light, photon energy is captured by the antennas inside the chloroplasts [2]. Part of the captured photons will be transferred forward for photochemical reactions [2,3], and the remainder will be emitted as heat or ChlF [4], which is often referred to as prompt fluorescence [5]. As the photochemical reactions involving plastoquinone Q_A_ and Q_B_ in Photosystem II (PSII) are partially reversible, some excited electrons may revert to P680 and be emitted as delayed fluorescence [6–8], but this emission is much weaker than prompt fluorescence and thus contributes little to measured ChlF [9]. Under high-light stress, heat is generated to prevent photooxidative damage [10–15] through nonphotochemical quenching (NPQ) [14–18]. The light-adaptation process is modulated by pH primarily through the xanthophyll cycle under high light [19–23] and regulated between the 2 photosystems under low light [24]. ChlF, photochemical quenching, and NPQ are energetically complementary, and ChlF can thus be measured to deduce photosynthetically important characteristics.

The commonly used ChlF magnitudes include those symbolized with F_0_, F_m_, and F_v_ for dark-adapted conditions and F_0_′, F_m_′, and F_v_′ for light-adapted conditions [25]. F_0_ represents the minimum fluorescence value when light is not sufficient to trigger photoelectron transport. F_m_ is the possible maximum fluorescence measured after application of a saturating pulse to an initially dark-adapted sample. The variable fluorescence, F_v_, is the difference between F_m_ and F_0_. Under light-adapted conditions, they are symbolized with F_0_′, F_m_′, and F_v_′, respectively. F′ is the steady-state fluorescence under an actinic light. These measured ChlF levels have allowed computation of various differences and ratios as measures of the PSII quantum efficiency (maximum or under actinic light), NPQ, photochemical quenching, and other aspects of PSII [26–29]. A major difficulty is that F_0_, F_0_′, and F′ are rather prone to variations in dark- or light-adaptation status and ambient lighting. Uncertainties in these important baseline levels can reduce the usefulness of the ChlF-based measures. Measurement of a magnitude or level often suffers from difficulties inherent in using an absolute scale. Dynamic responses to variable excitations could be an advantageous alternative for some applications but have rarely been explored.

In this research, we aimed to analyze the dynamic ChlF responses to time-variable excitations during strong actinic illumination. This initial attempt included observations in the time domain but focused on frequency-domain analysis. A pseudo-random binary sequence (PRBS) was designed and used as a multifrequency excitation illumination and dynamic ChlF responses were measured from different plant samples. Power spectrum analysis was used to compute the amplitude gains as functions of excitation frequency. Frequency-domain analysis revealed extra variations during strong actinic illumination and yielded a compensation method to reduce the effect of these variations in ChlF measurement.

## Materials and Methods

### Plant samples

*Spinacia oleracea, Arabidopsis thaliana, Citrus mitis*, *Quercus palustris*, and *Jasminum officinale* plants were used. Fresh *S. oleracea* samples were acquired from a local market. The *A. thaliana* plants were grown in a greenhouse under natural lighting without any treatments. These 2 plants were chosen as they are commonly used on photosynthesis-related research. The *C. mitis* and *J. officinale* were potted plants with identification tags acquired from a local Lowe’s store and were kept under natural light. The *Q. palustris* was a tree grown outdoors in Columbia, MO and was identified by a US Department of Agriculture tree scientist. These outdoor or indoor woody plants were chosen to broaden the range of plants used. The plants were in normal growing conditions without visible diseases. The sample collection was carried out in accordance with relevant guidelines and laws. Five leaves were severed as replications each time for measurement. Except for the moisture loss test as described in a following subsection, fresh intact leaf samples were used for fluorescence measurement immediately after they were severed.

### Fluorescence measurement

ChlF was measured with a modulated chlorophyll fluorometer (Model OS5p^+^, Opti-Sciences, Hudson, NH, USA) customized by the manufacturer for this research. The illumination, sensing and sampling mechanisms of the unit were not altered. The main changes were in the memory capacity and programmability to allow implementation of a time-variant excitation signal. To capture the variations in different measurement periods, ChlF was measured at 100-kHz sampling frequency for the first 0.1 s, then 100 Hz until 50 s, and 50 Hz during the PRBS variation as described below.

### Excitation signal

The excitation signal consisted of a wide strong actinic pulse followed by a PRBS of high and low light intensities as described below. The built-in white light-emitting diode actinic light of the OS5p^+^ instrument was used as the excitation source. Based on what was reported in the literature, a constant strong actinic light of 2,500 μmol/m^2^/s was first applied for 50 s (the wide pulse) to ensure commencement of adaptation activities before application of the PRBS signal [30,31]. The samples were initially dark-adapted in measurement clips for 30 min. The wide pulse thus allowed measurement of the initial OJIP phases of ChlF and a segment during adaptation to strong actinic illumination. The PRBS then followed to generate multifrequency excitation. Details of PRBS design can be found in many references [32,33]. The unit pulse width, the number of registers, and the logic polynomial for the PRBS were selected so that the excitation energy would be concentrated between 0.004 and 2π rad/s. From prior research, ChlF varied mainly under 2π rad/s [34]. The low limit was experimentally determined so that slow variations associated with strong actinic illumination or NPQ activities could be observed.

The mean level of the PRBS signal was chosen to equal to the intensity of the initial constant actinic light so that the PRBS signal would add high-frequency or rapidly changing perturbations without changing the mean illumination level at 2,500 μmol/m^2^/s. The low and high light intensity levels of the PRBS were 1,000 and 4,000 μmol/m^2^/s, respectively.

To explore the effect of the PRBS amplitude, tests were conducted for 2 additional amplitudes without changing the mean illumination level. The high and the low intensities of the PRBS signal were 500 and 4,500 μmol/m^2^/s, respectively, for a high-amplitude test and 1,500 and 3,500 μmol/m^2^/s, respectively, for a low-amplitude test. The illumination intensities were chosen to avoid extreme conditions based on ranges and values reported in the literature.

### Moisture content measurement

Moisture loss was used to induce changes in the photoenergy harvesting and transporting process in the samples and to observe if the analysis remained valid under moisture-stressed conditions. The moisture loss was indicated by the commonly used relative water content (RWC), which is (sample weight − dry weight)/(turgid weight − dry weight) × 100. The samples were first dipped in water for at least 8 h to reach turgid weight and pressure. The samples were then weighed and subjected to ChlF measurement while they progressively lost moisture in room conditions (22 °C, 30% relative humanity, and fluorescent lighting). Finally, the samples were dried in an oven for 6 h to obtain the dry weight. The samples were weighed with a precision balance (Model PX323/E, Ohaus, Parsippany, NJ, USA).

### Data analysis

Matlab (Version R2018a, MathWorks, Natick, MA, USA) was used for frequency analysis of the measured ChlF in response to the PRBS excitation. Power spectral analysis was performed on the temporal light-intensity variations (note: not the spectra of the electromagnetic waves) of the PRBS excitation signal and the corresponding ChlF response, both of which are time sequences as shown in Fig. 2. As described in books such as [35], a transfer or gain function from excitation to response can be computed for 2 time sequences as

$$G\left(\omega \right)=\frac{S_{\mathrm{uy}}\left(\omega \right)}{S_{u}\left(\omega \right)}$$ (1)

where *S_uy_* is the cross power spectrum or cospectrum between the excitation (PRBS illumination intensity) and the response (measured ChlF), *S_u_* is the power spectrum of the excitation itself, and *ω* is frequency. Since the spectra are functions of frequency, *G*(*ω*) is a frequency-dependent complex quantity. The magnitude or absolute value of *G*(*ω*) is commonly referred to as the excitation-to-response *gain* and is the amplitude ratio of response over excitation when the excitation is decomposed into different Fourier harmonic or sinusoidal components of different frequencies such as cos(*ωt*). The low-frequency gain thus indicates the strength of response to slow changes (including constant) in excitation and the high-frequency gain shows that to fast changes in excitation. This amplitude gain was computed in Matlab and analyzed in this work to reveal dynamic characteristics of ChlF in the frequency domain (or as functions of frequency).

**Fig. 1.**
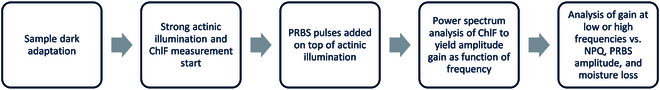
Flowchart for the experiment and data analysis process.

The ANOVA and Regression functions in Excel (Version 16.49, Microsoft, Bellevue, WA, USA) were used for group mean comparison and regression analysis, respectively. Differences were compared by Tukey’s multiple comparison with a significance level of *α* = 0.05. The moisture test data for all 5 samples in each set were pooled for regression of amplitude gain vs. RWC with a quadratic polynomial. The experiment and analysis process is summarized in the flowchart in Fig. 1.

## Results and Discussion

### Observation of NPQ effects on ChlF responses in the time domain

**Fig. 2.**
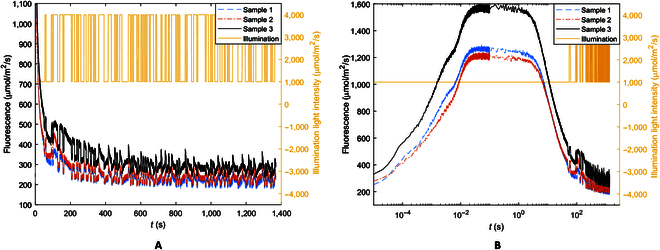
ChlF responses of fresh *S. oleracea* samples to a strong actinic illumination followed by a PRBS illumination signal. (A) Normal time scale. (B) Logarithmic time scale for clarity of the early OJIP phases of the responses.

Figure 2 shows the ChlF responses of initially dark-adapted *S. oleracea* samples to a wide pulse (50 s at 2,500 μmol/m^2^/s) followed by a PRBS signal (only 3 samples are shown for clarity and measurements from other samples were similar). The responses to the wide pulse followed a known pattern, rising initially through what is commonly referred to as the OJIP phases and then falling through what is known as the PSMT phase [36,37] on which the responses to the PRBS signal were superimposed. Beyond the peak point (usually labeled as P), ChlF starts to decline as a result of photochemical quenching initially and then light-adaptation or NPQ activities [38]. While this general pattern is familiar, several observations may be readily made about the effects of NPQ on ChlF responses.

When replicate samples from the same plant or the same leaf cluster of a plant were compared, their responses differed though the samples were presumably in similar, if not identical, conditions. First, the ChlF responses reached different peak values (see Fig. 2B). More importantly, after activation of NPQ mechanisms beyond the peak, ChlF descended in the PSMT phases at different rates, leading to a slow and unequal mean (or bias) in the responses to the PRBS signal. As Fig. 2B shows, sample 1 had a higher peak and reduction rate than sample 2. Sample 3 had a similar reduction rate to the other 2 samples but a higher peak and higher final stable average ChlF. This shows that even among replicated samples, there are not consistencies in the peak fluorescence, at which NPQ initiates, and in the rate and amount of ChlF reduction in the PSMT phases resulting from light adaptation.

From Fig. 2A, it can be seen that as adaptation progresses and the overall ChlF level trends down, the amplitude of the responses to the PRBS excitation decreases. This reduction is more discernable in Fig. 4A shown later. This makes sense because light adaptation activities decrease photon capture while increasing NPQ and heat generation, which will reduce photons reemitted as ChlF. This amplitude modulation effect, however, would complicate the observation of ChlF kinetics associated with photochemical reactions while adaptation is active since measured ChlF responses will be nonstationary in the sense that the amplitude will vary with time. This explains and is a manifestation of the variabilities in measured F_0_′, F_m_′, and F_v_′ associated with variations in the degree of light adaptation [25].

**Fig. 3.**
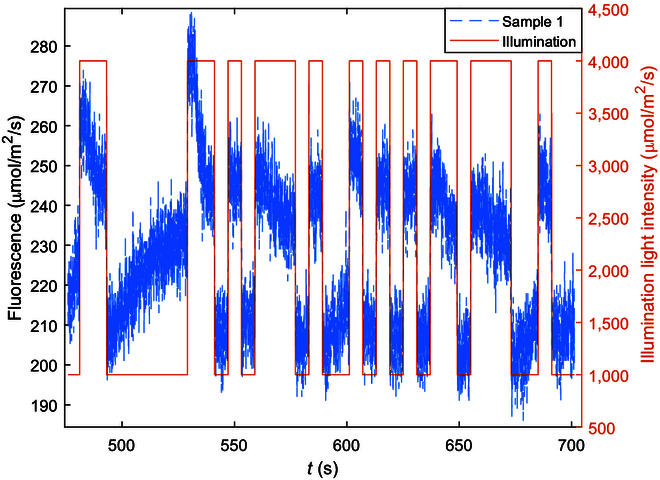
An enlarged view of ChlF responses to PRBS illumination during the intense illumination showing that the response decreased during a high excitation level and increased during a low excitation level (reverse response) after initiation of NPQ.

Figure 3 is a magnified view of the ChlF responses of one sample to the PRBS illumination. It can be noted that ChlF is largely “in phase” with the illumination in that it rises and falls with the rapid (or high-frequency) switching between the high and the low PRBS levels, which results in rapid changes in excessive light and thus in ChlF emissions. This is similar to how F_m_′ is measured with an excitation pulse. ChlF, however, continues to vary during a high- or low-pulse interval initially in the same direction as but mostly in the opposite direction to the PRBS level change (downward during high level and upward during low level) as observable during the wider pulses in Fig. 3. The same phenomenon is observed when a stronger actinic light is applied [25]. Such reverse changes in ChlF are indicative of negative feedbacks. This is consistent with the known mechanisms of light adaptation and photoprotection briefly summarized in the Introduction section [22,23].

These during-pulse variations are not symmetrical as seen in Fig. 3. A careful examination of all the samples shows that these during-pulse inverse variations or reversals are dependent on the illumination intensity and may or may not be obvious during a short interval. This is consistent with the fact that NPQ activities are nonlinear [17,18] and shows that NPQ activities are compounded with the ChlF responses to the PRBS perturbation in a nonlinear fashion. Interestingly, these reverse changes appeared as slow variations when illumination was constant (thus of low frequency) during a pulse following rapid changes (thus of higher frequency) after a step level change in excitation. This indicates an opportunity to analyze the responses in the frequency domain.

### Frequency-domain analysis

**Fig. 4.**
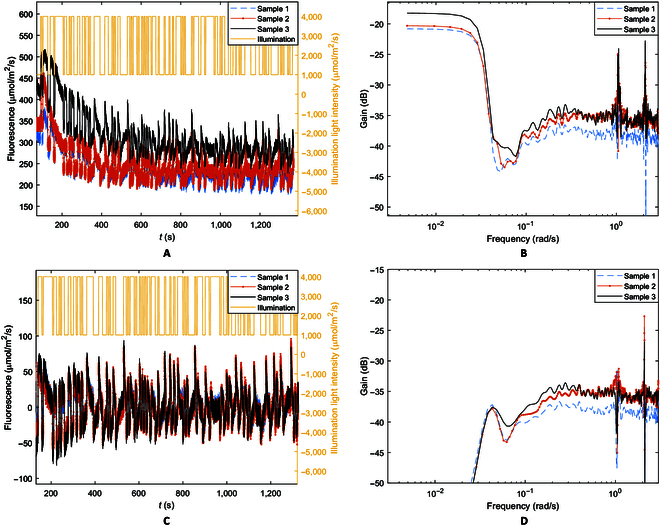
Frequency analysis of ChlF for *S. oleracea* samples. (A) ChlF responses to PRBS illumination excitation by *S. oleracea* samples. (B) Amplitude-gain plot based on the original ChlF measurement. (C) ChlF responses after detrending by high-pass filtering. (D) Amplitude-gain plot based on the detrended ChlF responses.

To build on the observation that strong actinic illumination appeared to affect ChlF changes in low frequencies, the ChlF responses to the PRBS excitation were analyzed in the frequency domain. Figure 4A replots the PRBS segment of the ChlF responses shown in Fig. 2, and Fig. 4B is the corresponding amplitude-gain (or ratio) plot of ChlF response over illumination excitation. There is an apparent correspondence between the amplitude gain in Fig. 4A and the level of the slow PSMT trend in the time domain (the mean or bias of the oscillatory responses in Fig. 4A), which indicates the extent of the intense illumination effect. A higher mean indicates a lower extent of energy diversion and heat generation by the NPQ mechanisms and consequently a higher amplitude gain in the ChlF response. This again is consistent with the fact that the pH-mediated light- adaption processes [21–23] are negative feedbacks which reduce the ChlF responses to illumination. It is important to note that the amplitude gain for both low and high frequencies shifted (Fig. 4B) corresponding to the mean ChlF levels in Fig. 4A, indicating that the intense illumination effect influences both the low- and high-frequency responses.

Figure 4B shows a steep gain drop around a cut-off frequency ω_c_ = 0.03 rad/s (or 0.01π rad/s). All the samples, including those of the other plant species tested (*C. mitis*, *Q. palustris*, and *A. thaliana*), consistently show this gain reduction at approximately the same frequency. The low-pass profiles (higher gain in low frequencies than in high frequencies) may be related to the slow downward trend in the PSMT phases observed in the time domain (Fig. 2A or 4A) resulting from actinic lighting [36,37].

For further analysis of the low-pass characteristic mentioned above, the ChlF responses were detrended by high-pass filtering to suppress frequencies below ω_c_ = 0.03 rad/s. The detrended time responses are shown in Fig. 4C and the amplitude gain plot in Fig. 4D. After filtering, the gain differences among samples in high frequencies remain largely unchanged (compare Fig. 4D with B). It is interesting to note that the filtering operation, which suppresses frequencies below 0.03 rad/s as shown by the gain diagram, largely detrends the time-domain responses and removes the nonzero mean (or bias) without significantly altering the high-frequency responses. This indicates that the amplitude gain below 0.03 rad/s resulted from the slow trend in the PSMT phases and thus the low-frequency gain may serve as an indicator of the extent of energy diversion by NPQ.

**Fig. 5.**
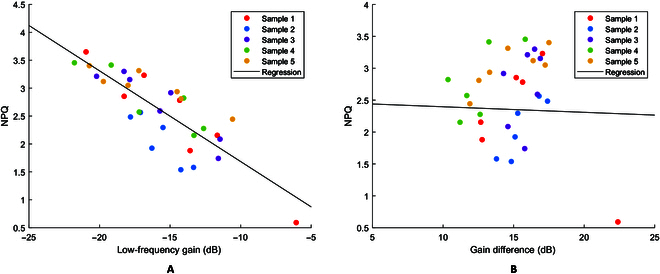
Regression of measured NPQ vs. gain. (A) NPQ vs. low-frequency gain (*R*^2^ = 0.66) and (B) NPQ vs. gain difference (*R*^2^ = 0.001) for *S. oleracea*.

To confirm that the low-frequency gain is an indicator of NPQ, Fm′ was measured with a saturation pulse of 7,000 μmol/m^2^/s during the same strong actinic illumination as that used in the PRBS measurements and NPQ was computed by using (Fm-Fm′)/Fm′. Regression results of the measured NPQ vs. low-frequency gain and gain difference for a *S. oleracea* sample are shown in Fig. 5A and B. The data show a negative linear relationship between NPQ and low-frequency gain and no apparent correlation between NPQ and gain difference. Each sample yielded similar linear negative correlations, but the slope and intercept of linear regression varied from sample to sample (Fig. 5A) as can be expected from the earlier discussions. When a regression line was fitted for each sample individually, the *R*^2^ values ranged from 0.721 to 0.892. This indicates that the low-frequency gain is linearly correlated with NPQ and may be used as a reference to compensate for the effects of variations in measured ChlF during strong actinic illumination as further tested and explored in the following sections.

### Excitation amplitude test

**Fig. 6.**
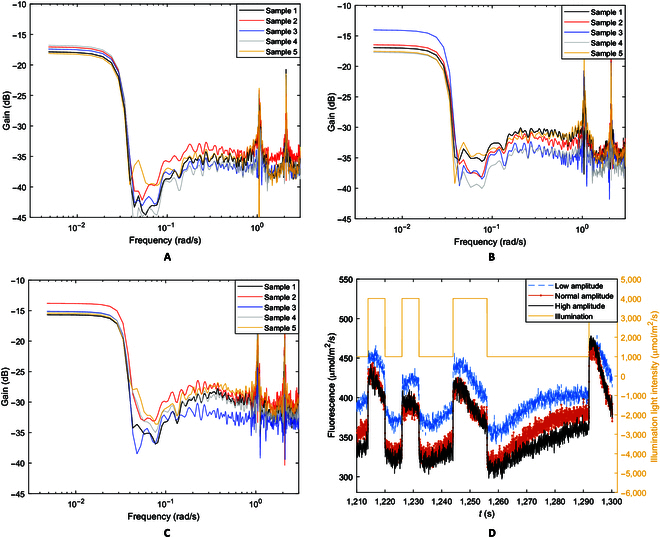
Amplitude-gain plots of *S. oleracea* samples for different PRBS excitation amplitudes. (A) 500 to 4,500 μmol/m^2^/s, (B) 1,000 to 4,000 μmol/m^2^/s, and (C) 1,500 to 3,500 μmol/m^2^/s. (D) ChlF responses to different excitation amplitudes in the time domain (only one excitation signal is shown). In response to the 3 exaction amplitudes, the average low-frequency gain between 0.004 and 0.01 rad/s did not differ significantly (*P* > 0.05) while the average high-frequency gain between 0.08 and 1.0 rad/s differed significantly (*P* < 0.05).

As a further observation of influences of light-adaptation activities on ChlF kinetics in the low frequencies, the amplitude of the PRBS excitation signal was varied while the initial wide pulse and the PRBS mean remained unchanged. Figure 6A to C shows the amplitude gain plots of *C. mitis* samples for 3 different PRBS amplitudes and Fig. 6D is the measured ChlF responses as functions of time, which show similar overall PSMT trends to those in Figs. 2 and 4, but the response amplitude varied with the excitation amplitude. From the graphs, it can be observed that when the amplitude of illumination changes, the general shape of the gain curves (Fig. 6A to C and the low-frequency gain values do not change greatly. The cutoff frequency ω_c_ remains at approximately 0.03 rad/s. The high-frequency gain, however, varies with the illumination amplitude. Tukey’s multiple comparison shows that the high-frequency gains are significantly different (*P* < 0.05) among the 3 excitation amplitudes while the low-frequency gains are not (*P* > 0.05). This indicates that the ChlF kinetic system is nonlinear because the gain, at least in high frequencies, varies with excitation amplitude (the gain does not vary with excitation amplitude for linear systems). This explains and supports the observed dependence of measured F_m_′ (and consequently F_q_′ and F_v_′) on the excitation pulse used and the effect of strong actinic illumination [25]. Interestingly, the low-frequency gain did not change significantly because the wide strong actinic pulse and the mean of the PRBS signal, which influence adaptation to strong illumination, were not changed in the experiments, again confirming that the low-frequency gain reflects NPQ.

### Moisture loss test

Plant water status has strong influences on ChlF response [39–41]. Moisture loss is known to affect photoelectron transport and thus photochemical reactions [42]. It may also change the NPQ activities of plants. To test if the observed low-frequency characteristics of the ChlF responses would hold when stress-induced changes occur, a series of measurements were made while the sample leaves progressively lost moisture under room conditions and until the coherence value between ChlF responses and excitation began to diminish, indicating that a sample has dried to a point where a measured fluorescence signal was no longer a response to a varying excitation. The progression of the moisture loss is indicated by the RWC.

**Fig. 7.**
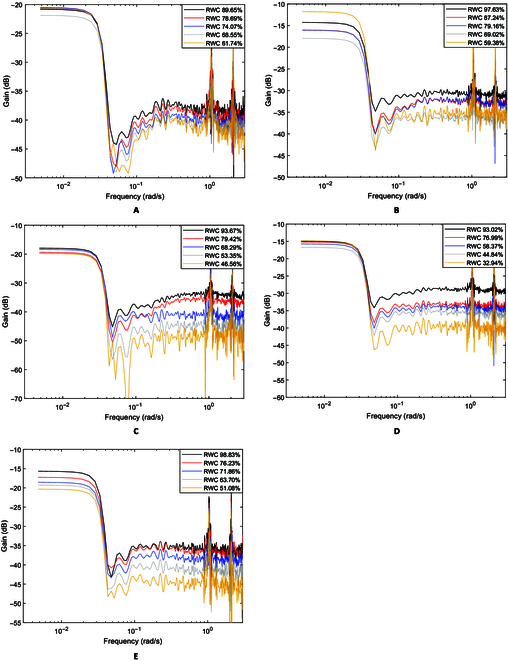
Amplitude gain plots of different species with gradual increase in sample moisture loss (decreasing RWC). (A) *S. oleracea*, (B) *C. mitis*, (C) *Q. palustris,* (D) *A. thaliana*, and (E) *J. officinale.*

Figure 7 presents the amplitude-gain plots of several *S. oleracea*, *C. mitis*, *Q. palustris,* and *A. thaliana* samples at different levels of moisture loss. As the graphs show, the low-pass shape of the curves remains with a cutoff frequency ω_c_ around 0.03 rad/s, and both the low-frequency and high-frequency gains vary among the samples. The gain of high frequencies between 0.08 and 1 rad/s generally decreases with increasing moisture loss (reducing RWC) in general but not always in the same order as RWC. For example, for *C. mitis* in Fig. 7B, the gain curve for 59.38% RWC is above rather than below that for a higher RWC of 69.02%. The inconsistencies are apparently associated with the inconsistent effects of the strong actinic illumination, which influence the low-frequency gain. As shown by the time-domain responses in Fig. 4A, the rate of the slow PSMT decay obviously differed among even replicated samples, indicating a varying effect of strong actinic illumination. This supports the observation that ChlF measurements are susceptible to ambient lighting and other variations [22,25] since the effect of strong actinic illumination may vary. As the low-frequency gain is associated with the slow decay and NPQ, the differences in the low-frequency gains (e.g., Fig. 7B) may offer help.

To test how the gain values varied with moisture loss, regression analysis was performed with the percent RWC as input and one of 3 gain values as response: (a) average low-frequency gain between 0.004 and 0.01 rad/s, (b) average high-frequency grain between 0.08 and 1 rad/s, or (c) the gain difference between the average low- and high-frequency gains. The regression *R*^2^ values are shown in the Table.

**Table.** *R*^2^ values for predicting different gain values from RWC for *S. oleracea*, *C. mitis*, *Q. palustris*, *A. thaliana*, and *J. officinale* (*n* = 5)

^o^	*R*^2^ value				
Response variable	*S. oleracea*	*C. mitis*	*Q. palustris*	*A. thaliana*	*J. officinale*
Low-frequency gain	0.1919	0.317	0.161	0.314	0.398
High-frequency gain	0.7048	0.813	0.866	0.888	0.462
Gain difference	0.7615	0.841	0.884	0.821	0.565

The regression analysis shows that the high-frequency gain is more dependent on water loss than the low-frequency gain does, and the gain difference is most responsive to water loss. This implies several points: First, water loss predominantly affected the high-frequency grain. Second, the low-frequency gain changed with water loss but to a much lesser degree. Third, by taking the gain difference and thus removing the low-frequency gain as a baseline, the *R*^2^ value improved, which means that the low-frequency gain, while apparently being affected by water loss, accounts for certain variations in NPQ during adaptation under strong actinic illumination. There is only a minor improvement in *R*^2^ and even a small reduction for the *Q. palustris* and *A. thaliana* samples tested. This is apparently related to the fact that the low-frequency gains did not differ significantly (*P* > 0.05) within each of the 2 sets of samples, indicating that the effect of strong actinic illumination did not vary greatly during the water loss tests. As a result, using the low-frequency gain as a baseline did not lead to major improvements than using the high-frequency gain alone.

**Fig. 8.**
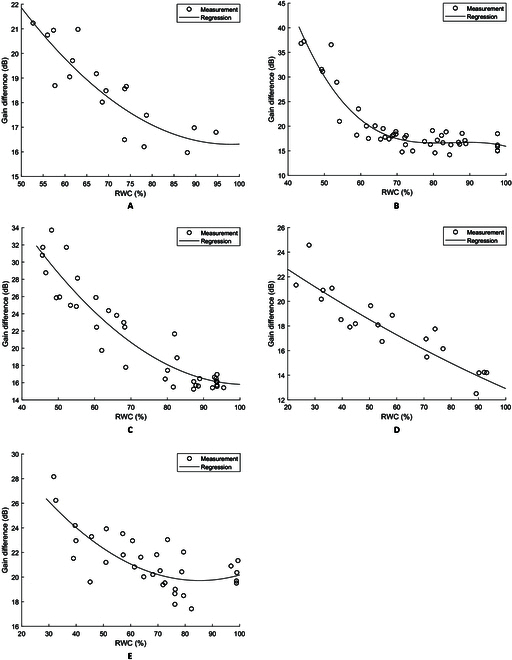
Example regression plots of gain difference vs. moisture loss. (A) *S. oleracea* (*R*^2^ = 0.7615, *P* < 0.05), (B) *C. mitis* (*R*^2^ = 0.880, *P* < 0.05), (C) *Q. palustris* (*R*^2^ = 0.884, *P* < 0.05), (D) *A. thaliana* (*R*^2^ = 0.821, *P* < 0.05), and (E) *J. officinale* (*R*^2^ = 0.565, *P* < 0.05).

Multiple water loss experiments were conducted, and the results were similar to those shown in the Table. With the gain difference as the response variable, the *R*^2^ values for *S. oleracea*, *C. mitis, Q. Palustris,* and *A. thaliana* fall in the ranges of [0.7259, 0.7561], [0.655, 0.881], [0.790, 0.915], and [0.810, 0.995], respectively. Example plots are shown in Fig. 8. By using the low-frequency gain to account for variations during adaptation to strong actinic illumination, the gain difference shows obvious dependence on water loss. Figure 8 shows that for the samples tested, the gain difference does not show obvious changes until RWC decreases to 80% to 85%. Both indoor and outdoor plants have a relationship between RWC and gain difference.

While additional experiments, especially field experiments, are needed, the data showed that the gain values from dynamic ChlF analysis exhibited similar properties to what some of the conventionally defined ChlF measures indicate, as suggested by the dynamic gain dependence on NPQ and moisture. A higher gain indicates a stronger ChlF response and thus greater values of commonly used measures such as F_m_ and F_m_′. Further research is clearly warranted.

To remove or separate the additional variations in ChlF measurements under strong illumination and to understand the molecular mechanisms, a mathematical model based on further research is ultimately needed for the light-adaptation kinetics. Until such a model is available, the low-frequency gain, as demonstrated in this initial research, can be used as a reference to improve ChlF measurements by, at least partially, removing variations related to varied degrees of adaptation.

## Conclusion

This research demonstrates that dynamic measurement and analysis can offer an additional avenue to enhance the use of ChlF measurement in plant research. Strong actinic illumination appears to influence ChlF more strongly in low frequencies, most notably below 0.03 rad/s. The low-frequency gain is linearly correlated with NPQ and showed usefulness as a reference to suppress variations in ChlF measurements. Strong actinic illumination affects the amplitude of ChlF and make the ChlF response to illumination excitation nonlinear and nonstationary. The amplitude-gain difference between low and high frequencies showed a strong dependence on plant water loss.
